# Children's acceptance of milk with xylitol or sorbitol for dental caries prevention

**DOI:** 10.1186/1472-6831-5-6

**Published:** 2005-07-22

**Authors:** Jorge L Castillo, Peter Milgrom, Susan E Coldwell, Ramon Castillo, Rocio Lazo

**Affiliations:** 1 Departamento Academico de Estomatología del Niño y del Adolescente, Universidad Peruana Cayetano Heredia, Honorio Delgado 430, Lima 34, Peru; 2 Dental Public Health Sciences, Box 357475, University of Washington, Seattle, USA 98195-7475; 3 Dental Public Health Sciences, Box 357475, University of Washington, Seattle, USA 98195-7475; 4 Departamento Academico de Estomatología del Niño y del Adolescente, Universidad Peruana Cayetano Heredia, Honorio Delgado 430, Lima 34, Peru; 5 Departamento Academico de Estomatología del Niño y del Adolescente, Universidad Peruana Cayetano Heredia, Honorio Delgado 430, Lima 34, Peru

## Abstract

**Background:**

Xylitol, a polyol sugar, has been shown to reduce dental caries when mixed with food or chewing gum. This study examines the taste acceptability of xylitol in milk as a first step toward measuring the effectiveness of xylitol in milk for the reduction of dental caries in a public health program.

**Methods:**

Three different types of milk (Ultra High Temperature (UHT), powder and evaporated) were tested for acceptability by 75 Peruvian children (25 per milk group, ages 4 to 7 years). Each group evaluated xylitol and sorbitol in one type of milk. In the first phase, each child was presented with a tray of four plastic cups containing 50 ml of milk with 0.021 g/ml xylitol, 0.042 g/ml xylitol, 0.042 g/ml sorbitol or no sugar. Each child was asked to taste the samples in a self-selected order. After tasting each sample, the child placed the milk cup in front of one of three cartoon faces (smile, frown or neutral) representing the child's response to the taste of each sample. In the second phase, the child was asked to rank order the milk samples within each category (smile, frown or neutral). Ranks within categories were then combined to obtain a rank ordering for all the test samples.

**Results:**

The ranking from best to worst for the samples across categories (UHT, powder, evaporated) was xylitol (0.0.042 g/ml), sorbitol (0.042 g/ml), xylitol (0.021 g/ml) and milk alone (Friedman's ANOVA). Xylitol and sorbitol were preferred over milk alone, and xylitol (0.042 g/ml) was preferred to sorbitol (0.042 g/ml)(p < .05 sign test).

**Conclusion:**

Milk sweetened with xylitol is well accepted by Peruvian children ages 4–7 years.

## Background

Xylitol is a non-nutritive sweetener that has demonstrated effectiveness for preventing dental caries [1]. It has been introduced in different foods for children including gum, candies, gelatin, sorbets, syrups and other products including multivitamins, lozenges, toothpaste, and oral rinses. Studies have demonstrated that the daily ingestion of 5 to 10 g of xylitol in different vehicles can reduce the levels of dental caries up to 82% [2-6]. This reduction can be explained by the effect of xylitol on cariogenic bacteria [7].

Xylitol has the same sweetness and bulk of sucrose but with one third fewer calories, approximately 2.4 cal/g [8]. Snacks made with xylitol are generally well accepted in preschool children and its application may be suitable for public health programs [9].

The perception of flavors in milk is one of the human infant's earliest sensory experiences, and there is support for the idea that this early experience with flavors has an effect on milk intake and on later food acceptance [10]. There have been attempts to use milk as a vehicle for caries prevention. While milk per se may have some protective action against dental caries [11], evidence suggests its impact is negligible to low if consumed in normal amounts [12]. Milk supplemented with fluoride has reduced dental caries rates in studies in Chile [13] and Hungary [14]. However, adding fluoride to milk in underdeveloped countries is potentially a problem because of the risk of overdose and fluorosis.

In Peru, chronic malnutrition is around 22 percent in children younger than five years old, but in children living in extreme poverty, it increases as high as 40 percent [15]. The Food and Agriculture Organization of the United Nations minimum recommended daily caloric intake for children is 2492 cal, but in Peru the part of the population living in extreme poverty consumes, on average, around 1658 cal. [16] In order to overcome this problem, there is a public health program called *El Vaso De Leche *(The Glass of Milk). This program offers a daily ration of food and milk to vulnerable populations, in order to improve their nutrition. The community actively participates in this program, always supported by the municipalities or the central government. Each child receives a daily glass of milk as part of the breakfast for the five weekdays. Among those who receive some type of food aid from the government, seventy eight percent belong to the *El Vaso De Leche *program [15].

Milk supplemented with xylitol has not been studied as a public health measure. If it can be demonstrated that xylitol added to milk has a beneficial anti-caries effect, it can be an important preventive measure in the young population, especially for children from low-income families who receive a daily glass of milk as part of the social programs in Peru. The purpose of this study is to examine the taste acceptability of xylitol in milk as a first step prior to measuring the effectiveness of xylitol in milk for the reduction of dental caries in a public health program.

## Methods

### Subjects

The parents of 108 out of 450 children (ages 4 to 7 years) enrolled in a primary school in Lima, Peru were approached in person and asked for permission to have their child participate in the study. Of the 108 parents contacted 101 (95%) agreed to allow their child to participate and 75 were included. Written, informed consent was obtained from these parents. Oral assent was also obtained from all the participating children prior to the initiation of study procedures. Study procedures were reviewed and approved by the human subjects committee at the Universidad Peruana Cayetano Heredia.

The study was carried out in three replications, one for each of the different types of milk (Ultra High Temperature (UHT), evaporated, or powdered) available in Peru. Twenty-five children participated in each replication, and no child participated in the study more than once. Ages of the children were matched across replications.

### Testing procedure

Taste testing was based on the two-part procedure of Birch and Sullivan [17]. This ranking procedure has been demonstrated to be reliable and valid in children three years of age and older [18,19].

Children were tested individually in the dental room of the school. Each child was presented with a tray containing four 120 ml transparent plastic cups filled with 50 ml of milk at room temperature. Location of each of the four choices on the tray was randomly determined. Cups were labeled with three-digit codes on the bottom of the cup in order to allow the experimenter to identify each item. The milk in each cup contained one of four formulations of milk, plain milk, milk with 0.021 g/ml xylitol (low xylitol), milk with 0.042 g/ml xylitol (high xylitol) or milk with 0.042 g/ml sorbitol. The doses of xylitol were chosen based on the amounts used in previous studies and assuming a daily consumption volume of 240 ml of milk with either 5 mgs or 10 mgs of xylitol or sorbitol in it (5 mg/240 ml = 0.021 mg/ml, 10 mg/240 ml = 0.042 mg/ml) [2,4,7].

Hedonic ratings for each milk formulation were obtained in the first phase of testing. Each child was asked to taste each of the four formulations of milk in a self-selected order. After tasting each milk sample, the child was asked to place the cup in front of one of three cartoon faces. The faces were a smile (like), a frown (dislike), or neutral expression. These faces were introduced to the children ahead of time in a practice session as indicating foods they like a lot (for example, a fruit juice), feel neutral about (water) or dislike (sugarless and cold tea). The hedonic category in which the child placed each milk sample was recorded.

Rankings of each of the four milk formulations were obtained in the second phase of testing. All the milks placed in the like category were presented to the child, and he/she was asked to taste them again and pick which one he/she liked the best. This milk was then removed, and the child was asked to taste the remaining items and asked again which one he/she liked the best. This was repeated with all remaining items in the like category. The procedure was then repeated with the other two hedonic categories. In this way a ranking of the four milks was achieved in a manner that was easy for young children. Rankings of the milk (first, second, third and fourth) were recorded by the investigators.

### Analysis

Friedman's ANOVA by ranks followed by post hoc sign tests were used to assess whether children's ranking of milks differed between formulations. Analyses were conducted separately for each study replication (UHT, evaporated, and powdered milk).

## Results

In all three milk types (UHT, powdered and evaporated), the 0.042 g/ml xylitol formulations were preferred to the other milks tested (χ^2^'s > 30, p < 0.00001, Z's > 2.0, p's < 0.05, Tables 2,3,4). Furthermore in all three milk types, xylitol 0.042 g/ml and sorbitol 0.042 g/ml were preferred to plain milk, and xylitol 0.042 g/ml was preferred to sorbitol 0.042 g/ml (Z's > 2.0, p's < 0.05, Tables 2,3,4).

**Table 2:** Children's ranking of UHT milks collapsed across preference categories

FOOD ITEM	1^ST^	2^ND^	3^RD^	4^TH^	MEDIAN RANK OF FOODS
XYLITOL 0.042 g/ml^a^	19	3	2	1	1
XYLITOL 0.021 g/ml^b^	3	8	10	4	3
SORBITOL 0.042 g/ml^c^	2	12	5	6	2
MILK ALONE^d^	1	2	8	14	4

Friedman ANOVA, N = 25, df = 3, Chi Square = 30.6, p < .00001. Food items labeled with different letters differ significantly from each other by sign test (p < 0.05).

**Table 3:** Children's ranking of powdered milks collapsed across preference categories

FOOD ITEM	1^ST^	2^ND^	3^RD^	4^TH^	MEDIAN RANK OF FOODS
XYLITOL 0.042 g/ml^a^	18	3	1	3	1
XYLITOL 0.021 g/ml^b^	1	8	13	3	3
SORBITOL 0.042 g/ml^c^	6	12	6	1	2
MILK ALONE^d^	0	2	5	18	4

Friedman ANOVA, N = 25, df = 3, Chi Square = 36.1, p < .000001. Food items labeled with different letters differ significantly from each other by sign test (p < 0.05).

**Table 4:** Children's ranking of evaporated milks across preference categories

FOOD ITEM	1^ST^	2^ND^	3^RD^	4^TH^	MEDIAN RANK OF FOODS
XYLITOL 0.042 g/ml^a^	21	2	0	2	1
XYLITOL 0.021 g/ml^b,c^	1	8	8	8	3
SORBITOL 0.042 g/ml^b^	2	12	9	2	2
MILK ALONE^c^	1	3	8	13	4

Friedman ANOVA, N = 25, df = 3, Chi Square = 33.7, p < .000001. Food items labeled with different letters differ significantly from each other by sign test (p < 0.05).

## Discussion

The purpose of this study was to determine if children would accept milk sweetened with xylitol. We used three different types of milk (UHT, powder and evaporated) because there is variation in the milk given to children in the social programs in Peru. All children completed the experiment, including four (2 UHT, 1 evaporated and 1 powder) who stated before the experiment that they did not like milk. Because excluding these children could have reduced the practical value of our the results of our study, they were included [20]. Moreover, a dislike of milk altogether is not likely to have had much influence on the results because the primary dependent measure of interest was the relative ranking of each milk type.

Most previous studies that have investigated the addition of xylitol to different types of food have demonstrated that five to 10 mg of xylitol affects *S. mutans *and reduces dental caries levels [4,6,21-23]. The doses we used (0.042 g/ml and 0.021 g/ml), resulted in either five or 10 mg of xylitol per glass.

Children preferred the milk with xylitol 0.042 mg/ml over milk with sorbitol 0.042 mg/ml. We informally asked some of the children what they found different between the milks. Some of the children stated that sorbitol tasted more artificial, and that xylitol was sweeter. Xylitol is the only sugar alcohol that has the same sweetness as sucrose [24]. Sorbitol is 40 percent less sweet that xylitol and sucrose [24]. Maltitol is the next closest in sweetness to sucrose (20 percent less sweet) [24].

This project is the first step towards a more comprehensive study that will look at the effects of the ingestion of milk with xylitol on cariogenic bacteria and on dental caries levels. One issue for a larger study is whether the properties of xylitol make it suitable to place it in milk. Xylitol forms very loose complexes with calcium under some conditions in vitro and delays precipitation of calcium; however, these features should have no impact on the bioavailability of the xylitol in milk [25]. Also, previous studies using other xylitol vehicles have had daily frequencies up to five. Studies are needed to ascertain whether single dose per day is substantive enough to modify the bacteria flora.

Overall, milk with xylitol is well accepted by children four to seven years old. If we can demonstrate that there is a relevant effect of xylitol in milk on cariogenic bacteria and levels of dental caries, as xylitol does in chewing gum, puddings, and other meals, we will have another public health tool to be applied in populations with high levels of the disease, and who have the availability of at least one glass of milk at school. In Peru, with programs like *El Vaso De Leche *that are directed to improve the nutrition of populations living under conditions of poverty, children may have an additional benefit if we can demonstrate that xylitol in milk has an anti-caries effect.

## Conclusion

This preliminary study found that xylitol in milk is acceptable to Peruvian children and that both xylitol and sorbitol in milk at 0.042 g/ml are preferred to plain milk. Xylitol is preferred over sorbitol.

## Competing interests

The author(s) declare that they have no competing interests.

## Authors' contributions

Dr. Castillo organized and directed the study. He served as the liaison to the schools and also wrote the initial draft of the manuscript. Drs. Milgrom and Coldwell contributed to the design, statistical analysis, and writing of the subsequent drafts. Drs. Castillo and Lazo carried out the field aspects of the study. All authors read and approved the final manuscript.

**Table 1:** Design of the milk groups presented to groups of children

GROUP 1 UHT N = 25	GROUP 2 POWDER N = 25	GROUP 3 EVAPORATED N = 25
0.021 g/ml XYLITOL	0.021 g/ml XYLITOL	0.021 g/ml XYLITOL
0.042 g/ml XYLITOL	0.042 g/ml XYLITOL	0.042 g/ml XYLITOL
MILK ALONE	MILK ALONE	MILK ALONE
0.042 g/ml SORBITOL	0.042 g/ml SORBITOL	0.042 g/ml SORBITOL

## Pre-publication history

The pre-publication history for this paper can be accessed here:



## References

[B1] Hayes C (2001). The effect of non-cariogenic sweeteners on the prevention of dental caries: a review of the evidence. J Dent Educ.

[B2] Isokangas P, Alanen P, Tiekso J, Makinen KK (1988). Xylitol chewing gum in caries prevention. A field study in children at caries-active ages. J Am Dent Assoc.

[B3] Isokangas P, Alanen P, Tiekso J, Makinen KK (1989). Long-term effect of xylitol chewing gum on dental caries. Community Dent Oral Epidemiol.

[B4] Makinen KK, Bennett CA, Hujoel PP, Isokangas PJ, Isotupa KP, Pape HR, Makinen PL (1995). Xylitol chewing gums and caries rates: A 40-month study. J Dent Res.

[B5] Hujoel PP, Makinen KK, Bennett CA, Isotupa KP, Isokangas PJ, Allen P, Makinen PL (1999). The optimum time to initiate habitual xylitol gum-chewing for obtaining long-term caries prevention. J Dent Res.

[B6] Alanen P, Isokangas P, Gutmann K (2000). Xylitol candies in caries prevention: results of a field study in Estonian children. Community Dent Oral Epidemiol.

[B7] Roberts MC, Riedy CA, Coldwell SE, Nagahama S, Judge K, Lam M, Kaakko T, Castillo JL, Milgrom P (2002). How xylitol-containing products affect cariogenic bacteria. J Am Dent Assoc.

[B8] Lindley MG, Birch GG, Khan R (1976). Sweetness of sucrose and xylitol. Structural considerations. J Sci Fd Agric.

[B9] Lam M, Riedy CA, Milgrom P, Coldwell SE, Craig R (2000). Children's acceptance of xylitol-based foods. Community Dent Oral Epidemiol.

[B10] Birch LL, Fisher JO (1998). Development of eating behaviors among children and adolescents. Pediatrics.

[B11] Bowen WH, Pearson SK (1993). Effect of milk on cariogenesis. Caries Res.

[B12] Grenby TH, Andrews AT, Mistry M, Williams RJ (2001). Dental caries-protective agents in milk and milk products: investigations in vitro. J Dent.

[B13] Marino R, Villa A, Guerrero S (2001). A community trial of fluoridated powdered milk in Chile. Community Dent Oral Epidemiol.

[B14] Scheinin A, Banoczy J, Szoke J, Esztari I, Pienihakkinen K, Scheinin U, Tiekso J, Zimmermann P, Hadas E (1985). Collaborative WHO xylitol field studies in Hungary. I. Three-year caries activity in institutionalized children. Acta Odontol Scand.

[B15] Gajate G, Inurritegui M (2003). El impacto del Vaso de Leche sobre el nivel de nutrición infantil. Economia y Sociedad.

[B16] Suarez MA Caracterización del programa del Vaso de Leche. http://www.mef.gob.pe/propuesta/ESPEC/caracvaso.pdf.

[B17] Birch LL, Sullivan SA (1991). Measuring children's food preferences. J School Health.

[B18] Birch LL (1979). Dimensions of preschool children's food preferences. J Nutr Educ.

[B19] Birch LL (1979). Preschool children's food preferences and consumption patterns. J Nutr Educ.

[B20] Chapman KW, Boor KJ (2001). Acceptance of 2% ultra-pasteurized milk by consumers, 6 to 11 years old. J Dairy Sci.

[B21] Soderling E, Makinen KK, Chen CY, Pape HR, Loesche W, Makinen PL (1989). Effect of sorbitol, xylitol and xylitol/sorbitol chewing gums on dental plaque. Caries Res.

[B22] Makinen KK, Soderling E, Isokangas P, Tenuovo J, Tiekso J (1989). Oral biochemical status and depression of Streptococcus mutans in children during 24- to 36- month use of xylitol chewing gum. Caries Res.

[B23] Isotupa KP, Gunn S, Chen C-Y, Lopatin D, Makinen KK (1995). Effect of polyol gums on dental plaque in orthodontic patients. Am J Orthod Dentofacial Orthop.

[B24] Bar A, Nabors LO, Gelardi RC (1992). Xylitol. Alternative Sweeteners.

[B25] Kennefick S, Cashman KD (2000). Investigation of an in vitro model for predicting the effect of food components on calcium availability from meals. Int J Food Sci Nutr.

